# Analysis of four *Echinococcus multilocularis* mitogenome sequences from Inner Mongolia, China: supporting the hypothesis that *E. sibiricensis* is confirmed as the O1 haplotype

**DOI:** 10.1186/s13071-025-07057-7

**Published:** 2025-11-04

**Authors:** Baoping Guo, Li Zhao, Zhe Cheng, Guodong Lü, Malike Aizezi, Liping Su, Gang Guo, Tongzhong Liu, Wanli Ban, Mehdi Borhani, Xinmiao Huang, Hao Wen

**Affiliations:** 1 State Key Laboratory of Pathogenesis, Prevention and Treatment of High Incidence Diseases in Central Asia, Clinical Medicine Institute, The First Affiliated Hospital of Xinjiang Medical University, No.137, Liyushan Road, Urumqi, Xinjiang People’s Republic of China; 2 Veterinary Research Institute, Xinjiang Academy of Animal Sciences, No. 726, Dongrong Street, Urumqi, Xinjiang People’s Republic of China; 3 State Key Laboratory of Cellular Stress Biology, School of Life Sciences, Xiamen University, Xiamen, Fujian People’s Republic of China; 4 Xinjiang Uyghur Autonomous Region Center for Animal Disease Control and Prevention, Urumqi, Xinjiang People’s Republic of China; 5 Xinjiang Clinical Research Center for Perinatal Diseases, Urumqi Maternal and Child Health Hospital, Urumqi, Xinjiang People’s Republic of China; 6 Xinjiang Autonomous Regional Center for Disease Control and Prevention, Urumqi, Xinjiang People’s Republic of China

**Keywords:** *Echinococcus multilocularis*, Inner Mongolia, China, *E. m. sibiricensis*, Mitochondrial genome (mitogenome), Phylogeny, Haplotype, *E. sibiricensis*

## Abstract

**Background:**

*Echinococcus multilocularis* is a zoonotic parasitic species that causes alveolar echinococcosis (AE), a severe disease affecting both humans and animals. This disease is particularly prevalent in the Northern Hemisphere, especially in northeast Asia, Europe, and North America. Previous studies have often conflated the haplotypes of *E. multilocularis* from Inner Mongolia, China, and Siberia, Russia. Furthermore, the unique variant identified in Inner Mongolia is of significant importance for elucidating the evolutionary history of *E. multilocularis*.

**Methods:**

The four complete mitochondrial genome (mitogenome) sequences obtained were amplified by polymerase chain reaction (PCR): one from an AE patient in Hulunbuir, Inner Mongolia, China, and three from *E. multilocularis* isolates maintained in gerbils at Academician Chongti Tang’s laboratory. Subsequently, these sequences underwent high-throughput sequencing using Illumina technology.

**Results:**

The four mitogenome sequences all span the full length of 13,738 base pair (bp). A phylogenetic analysis was conducted to assess the genetic differences between these sequences and others derived from major *E. multilocularis* endemic regions globally, with a particular focus on northeast Asia. The results demonstrated that the similarity among the four sequences was 99.24–99.26%. Furthermore, the genetic divergence between sequences originating from Mongolia, Siberia, Russia, and North America was relatively low, indicating a high degree of sequence similarity. The four sequences from Inner Mongolia, China were classified into four haplotypes: O1–O4. Sequencing and genetic analysis confirmed that the previously published *E. m. sibiricensis* belongs to the O1 haplotype.

**Conclusions:**

This study clarifies the genetic relationship between *E. multilocularis* haplotypes in Siberia, Russia, and Inner Mongolia, China, confirming that *E. m. sibiricensis* is part of the O1 haplotype. The findings strengthen the foundation for molecular epidemiology of AE and underscore the need for international collaboration in monitoring this zoonotic pathogen. Public health strategies can leverage these insights to predict and prevent outbreaks, particularly in endemic regions.

**Graphical Abstract:**

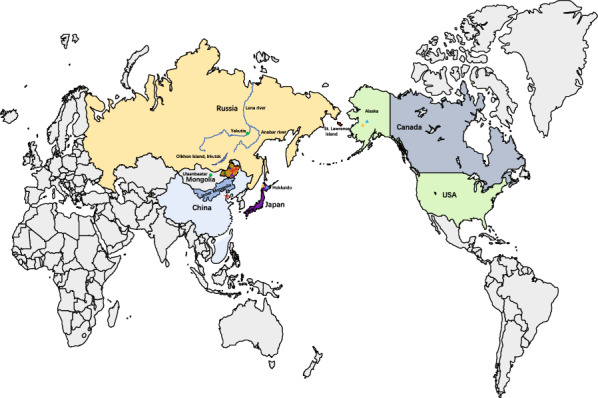

**Supplementary Information:**

The online version contains supplementary material available at 10.1186/s13071-025-07057-7.

## Background

Alveolar echinococcosis (AE) is a neglected zoonosis caused by the larval stage of the tapeworm *Echinococcus multilocularis*, which is found throughout the Arctic ecozone [1–3]. The endemic areas in Central Europe are not linked to those in Asia, and the Bering Strait is the dividing line between the North American and Asian endemic regions [4, 5]. Understanding the unique distribution requires consideration of the dispersal, isolation, and extinction of its mammalian hosts during the Pleistocene (1.8 million to 10,000 years ago) [6]. Recurrent glacial events during this period may have fragmented and migrated *E. multilocularis* populations. Red foxes (*Vulpes vulpes*) and arctic foxes (*Vulpes lagopus*) are definitive hosts, while various rodents from the Cricidae and Muridae families serve as intermediate hosts, integral to the parasite's life cycle in these wild animals [6–8].

Rausch and Schiller [9] identified *E. sibiricensis* in arctic foxes and sled dogs on St. Lawrence Island [10], Alaska, USA. *E. sibiricensis* is morphologically the same as *E. multilocularis* found in southern Germany, but it is considered a subspecies (*E. multilocularis sibiricensis*) because of biological differences, with both occurring in Inner Mongolia, China [11–13]. Historically, *E. multilocularis* has been classified into geographic subspecies, including *E. multilocularis* in Central Europe, *E. m. sibiricensis* in Alaska, and *E. m. kazakhensis* in Kazakhstan [3]. However, the geographic subspecies is primarily based on morphology and geographical distribution, which is insufficient for a comprehensive understanding. The geographic pattern of genetic variation in *E. multilocularis* was first characterized with the use of mitochondrial genome (mitogenome) sequences [14, 15]. Nakao et al. defined geographic clades as European (E1–E5), Asian (A1–A10), and North American (N1–N2) on the basis of phylogenetic trees and parsimony networks, noting that the Inner Mongolia haplotype (O1) was genetically different from other haplotypes [6]. The unique variant in O1–O4 is particularly valuable for studying the evolutionary history of *E. multilocularis*. Evolutionary analyses indicate a certain relationship between O1–O4, N1, and N2 in the host–parasite interrelationship, on the basis of concatenated sequences of the genes *cob*, *nad2*, and *cox1*.

Prior research has been limited to the analysis of concatenated sequences of three genes. A comprehensive analysis of the full-length mitogenome sequence from Inner Mongolia, China is currently lacking, with only one concatenated sequence previously reported. Furthermore, a full-length mitogenome sequence of a human infected with the O haplotype has been not found.

In this article, we report on the diversity of full-length mitogenomes for *E. multilocularis* haplotypes O1–O4. The full-length mitogenomes exhibit the following characteristics: (1) *E. multilocularis sibiricensis* is in fact *E. multilocularis* haplotype O1; (2) haplotypes O1–O4 are found to coexist in Inner Mongolia, China; (3) the relationships among haplotypes O and other haplotypes can be distinguished; and (4) notable genetic diversity exists within *E. multilocularis* haplotype O. This study provides a valuable framework for evaluating genetic diversity at both the interspecific and intraspecific levels. To generate the most comprehensive dataset possible, we sequenced the full-length mitogenomes of *E. multilocularis* haplotype O from each geographic isolate. The findings highlight how geographic characteristics and human activities shape the transmission dynamics of the zoonotic parasites, providing important insights for reducing the risk of potential future outbreaks.

## Methods

### Human samples

**Fig. 1:**
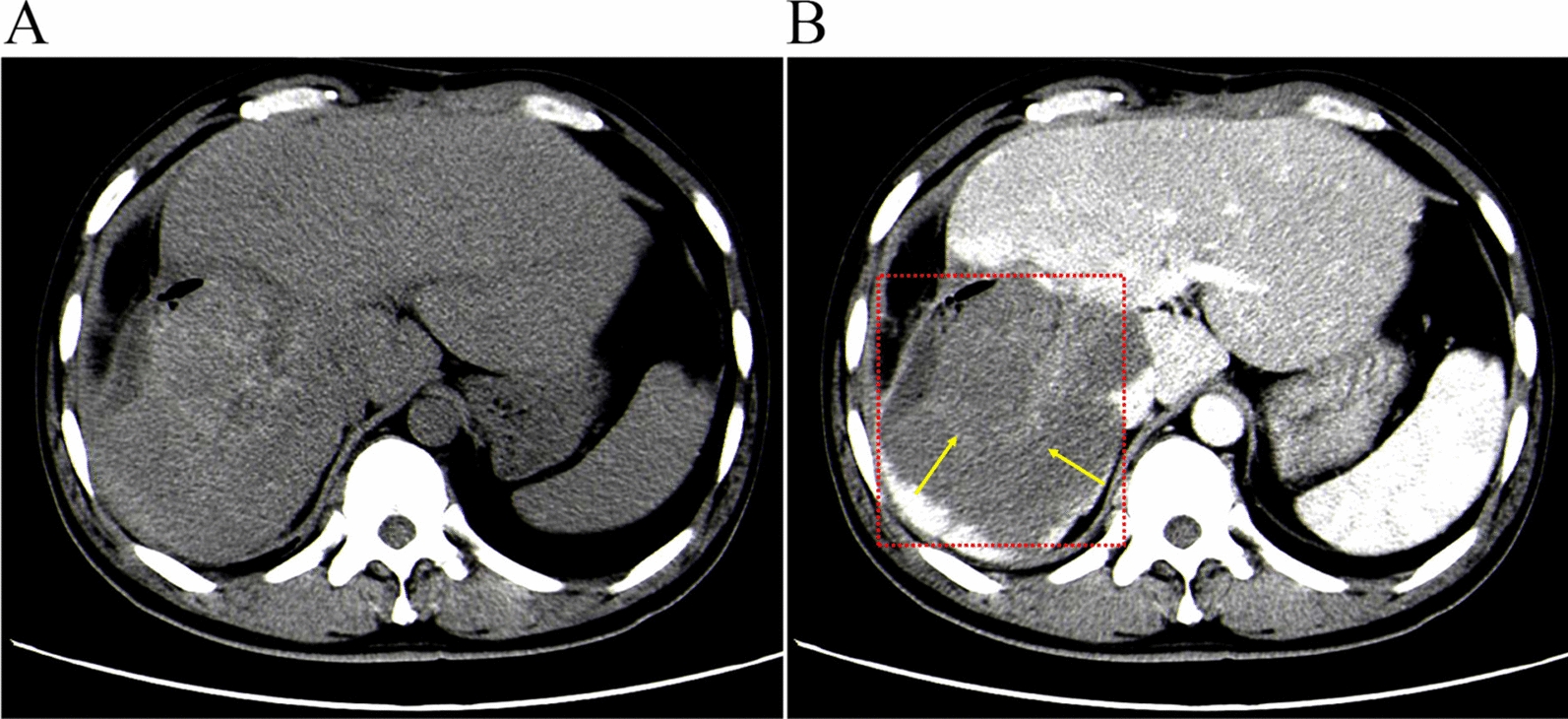
Imaging findings of liver lesions. **A** Nonenhanced scan image of the liver; **B** Enhanced scan image of the liver. Abdominal dynamic computed tomography (red arrow) revealed round low-density shadows in the right lobe of the liver, with no enhancement observed following the contrast-enhanced scan (yellow arrow). A mass of mixed density was identified on the right side of the liver, characterized by low soft tissue density, some calcification, and local hepatic indentation. A hybrid mass measuring 11.6 × 11.2 cm^2^ was noted in the right posterior lobe of the liver (red box)

On 8 June 2012, a 35 year-old man from Molidawa Dahan Autonomous Flag in Hulunbuir, Inner Mongolia, China presented to the Department of Hepatobiliary Echinococcosis at the First Affiliated Hospital of Xinjiang Medical University for treatment of severe upper abdominal pain. The patient had resided in the pastoral areas of Hulunbuir for an extended period and had previously enjoyed good health. A physical examination revealed a mass in the right upper abdomen measuring 17.0 × 13.0 cm^2^, which was accompanied by tenderness (Fig. 1). A computed tomography (CT) scan of the liver showed a round lesion approximately 12.5 × 7.3 cm^2^ in size [16, 17]. During surgery, the right upper abdomen was explored, and the lesion was found to measure about 23 × 16 cm^2^ in the right lobe of the liver, invading the biliary tract, diaphragm, duodenum, and colon. The left lobe of the liver was compensatorily enlarged, while the spleen, stomach, and intestines appeared normal. The patient’s surgical specimens were stored at –80 °C until further analysis.

### Protoscoleces samples

The three distinct *E. multilocularis* (M1–M3) protoscoleces (PSCs) were obtained from intraperitoneal lesions maintained in gerbils infected in Hulunbuir in 1998. Subsequently, the clean PSCs were provided by the research group of Academician Chongti Tang at Xiamen University and were counted according to the methods described previously [18]. Afterward, 10,000 PSCs from each sample were transferred into Eppendorf tubes.

### Extracted DNA

Genomic DNA was extracted from human liver lesion tissue (H1) and three PSCs using the CWBOI Universal Genomic DNA Kit (CWBOI, Beijing, China) according to the manufacturer’s instructions. DNA was eluted with 2 × 50 μl RNase-free water, centrifuged at 12,000 rpm for 2 min, and then stored at −20 °C until needed.

### PCR amplification

The mitogenome reference sequence of *E. multilocularis* used in this study was previously described [6]. The full-length mitogenome sequences of *E. multilocularis* were amplified using two overlapping PCR methods, with primers for the 11 fragments designed by aligning the mitogenome sequences of *E. multilocularis* (AB018440) deposited in the database (Table 1). For the mitogenome sequence, we designed 11 primer pairs using online software (https://www.bioinformatics.nl/cgi-bin/primer3plus/primer3plus.cgi). A 50 μL PCR reaction mixture was prepared, containing 2 μL genomic DNA, 25 μL 2 × PCR mixture (including PCR buffer, Taq DNA polymerase, and dNTPs from CW BOI, Beijing, China), 1 μL each of forward and reverse primers, and 21 μL of distilled water. Reaction conditions consisted of an initial denaturation step at 95 °C for 2 min, followed by 35 cycles for 30 s denaturation at 95 °C, annealing at 56 °C for 2 min, and extension at 72 °C for 2 min. A final extension was performed for 5 min at 72 °C. The PCR products were then sent to Anhui General Biological Co., Ltd. (Chuzhou, China) for Sanger sequencing.

**Table 1:** Primers designed on 11 fragments for the amplification and sequencing of the complete mitogenome of *Echinococcus multilocularis*

No	Forward primer (5′-3′)	Reverse primer (5′-3′)	Size (bp)
E1	TGTGTTGGTTAAAGTGGAGTTTAGAA	CAAACACAGTCAACAAAAGCA	1428
E2	GGGCCCCTACTCCAGTTAGT	CCAAAAATCGCTACTTCACTCA	1467
E3	GGTTCCTGTGTTTGGTGCTT	AAACAAGACTAAATCCTTAACAGAAAA	1383
E4	GTTGGTGGATTTTCGGTGTC	GAAACATAACCTTTATAACACCACTCA	1334
E5	TTTTTGATGTGTTTATGAATTATCG	TGGTGTACCTAACGGAACAAAA	1429
E6	TCTTGGGTGGTTTTTGGTTT	AGTAAAGCTCAAACCGAGTTCTCTC	1389
E7	TTTTGTTTAAGTTGGGTGGTGA	GAACTCAGCTCTTCGCAACA	1430
E8	TTTAAATCGTGATGCTGTTAACTT	CCAAAACAGGCAACGTCACT	1356
E9	TTTTCTTTGCATTTAGCAGGTG	CGACTTACTTATCACACCACAAATC	1403
E10	TGCCTTTTGCATCATGCTT	CCAGACATACACCAACATAAAAAGA	1457
E11	AAGATATATGTGGTGACAGGGATT	CCAAACACTAAAGTCAAACACAAA	1518

### Haplotype network analysis

In this study, the concatenated sequences of three genes: *cob* (1068 bp), *nad2* (882 bp), and *cox1* (1608 bp), a total of 3558 bp, were aligned with different sequences from Europe (E1–E5), Asia (A1–A10), and North America (N1–N2) [19, 20], and O1, as previously reported by Nakao et al. [6] (Supplementary Table S1). The full-length sequences in this research were also aligned with sequences from A1–A10, E1, and N1–N2, as documented by Hayashi et al. (Supplementary Table S2) [21]. Sequence analysis was conducted using BioEdit 5.0 software (http://www.mbio.ncsu.edu/bioedit/bioedit.html), and the sequencing results were further analyzed by sequence alignment (MAFFT comparison and NJ/UPGMA phylogenetic analysis) [22]. We performed BLAST analysis (http://www.ncbi.nlm.nih.gov/Blast.cgi) and compared the existing *cob*, *nad2*, and *cox1* sequences in the GenBank database. The mitogenome haplotype network was illustrated using PopArt 1.7 with statistical parsimony [23]. The network estimation was performed with a 95% connectivity limit, and haplotype frequencies in the network were not included in this study. Genetic distance was analyzed using the pairwise fixation index (F_ST_) with Arlequin 3.5.2.2 [24]. The software MEGA10 was employed to construct phylogenetic trees using the neighbor-joining (NJ) method. The NJ tree was constructed based on Kimura's two-parameter distances with a gamma shape parameter (*α* = 0.5). The robustness of the phylogenetic trees was assessed through bootstrapping with 1000 replicates.

### Phylogenetic analysis

Bayesian discrete phylogeography methods were used to infer the dispersal process of *E. multilocularis*. This approach labels tree nodes with the most likely ancestral positions and allows researchers to estimate phylogenetic diffusion processes using Bayesian random search variable selection (BSSVS) [25, 26]. Specifically, we utilized tandem sequences from three genes (*cob*, *nad2*, and *cox1*) and the full-length mitogenome sequences, assigning their respective sampling locations, as described above. All sequences were aligned using MAFFT v7.475 [22]. BEAUti v1.10.4 was used to generate the XML input file for BEAST v1.10.4 [27]. The best-fit model for nucleotide substitution was estimated using Kakusan4 [28, 29], and a generalized time-reversible (GTR) model was applied to estimate base frequencies and gamma heterogeneity across the four categories [30]. We conducted two independent runs of 400,000,000 generations, sampling every 2000 steps, and achieved effective sample size (ESS) values over 200 for all parameters concerning ucld and kappa. Substitution rates were estimated using a shorter BEAST run (chain length 10,000,000) and included as a priori data in the final analysis. The clock type was set to a slack clock, and *E. multilocularis* had a log-normal distribution. In both cases, the tree prior was configured as a Bayesian skyline to efficiently estimate population size. Convergence was checked using Tracer v1.6, and the number of samples removed as burn-in was determined. We merged the remaining logs and trees using LogCombiner v1.10.4, discarded the first 10% of each run, and generated a maximum clade confidence (MCC) tree using TreeAnnotator. The MCC tree was visualized using FigTree.

## Results

### Human histopathological morphology

**Fig. 2:**
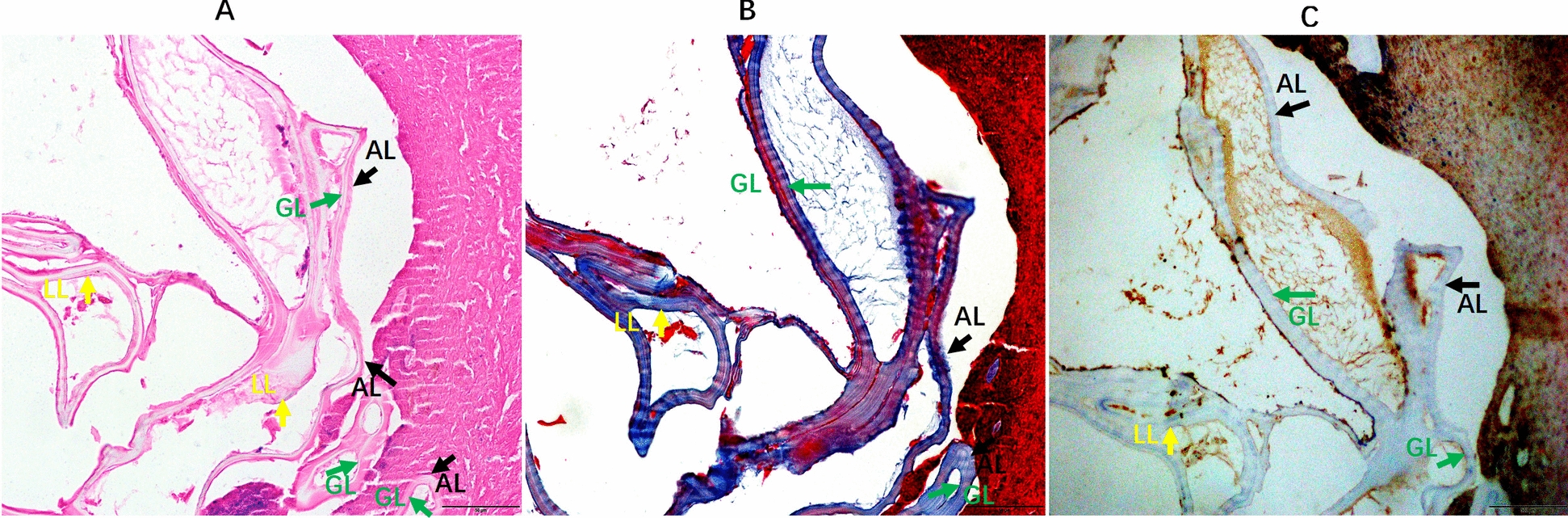
Pathological findings of human liver lesions. **A** The hematoxylin and eosin (H&E) stain of paraffin sections reveals distinct layers (× 200). Note: the black arrow indicates a cystic lesion, the black arrows denote the adventitial layer (AL), the green arrows highlight the laminated layers (LL), and the yellow arrows indicate the germinal layer (GL). **B** Liver fibrosis assessed by Masson’s staining (× 200). **C** Immunohistochemical (IHC) staining of α-smooth muscle actin (α-SMA) in the mouse liver (× 200). H&E, hematoxylin and eosin; α-SMA, α-smooth muscle actin. Pv, parasitic vesicle

Histopathology of liver tissue from resection with staining showed granulomatous inflammation. No PSCs were observed under a light microscope, and the parasitic lesions were not fertile according to hematoxylin and eosin (H&E) staining (Fig. 2A). However, the parasitic lesions were characterized by the presence of AL, LL, and GL (Fig. 2A–C). Masson staining showed inflammation and fibrosis due to liver infection (Fig. 2B). Immunohistochemical analysis revealed significant expression of α-smooth muscle actin (α-SMA) around the parasite foci (Fig. 2C) (Abcam, UK), consistent with previous reports [31, 32]. The formation of parasitic lesions followed the typical pattern of AL, LL and GL, and the number of parasitic lesions gradually increased after infection. A large number of fibroblasts and inflammatory cells were observed surrounding the parasitic lesions. Granulomas, consisting of inflammatory cells and fibrosis, suggest a typical pattern of *E. multilocularis* infection [33, 34].

### Mitogenome characterization

We analyzed four mitogenome sequences of *E. multilocularis*, including three from maintained gerbils and one from a patient. All four mitogenome sequences were 13, 738 base pairs (bp) in full length. During the analysis, full-length sequences of O1 and M1 were completely identical, but concatenated sequences of *cob*, *nad2*, and *cox1* from H1 when compared with sequences from northeast Asia (O1, M1, M2, M3, Mongolia, OR911453) were found to be 99.89%, 99.89%, 99.86%, 99.86%, 99.49%, and 99.54% identical, respectively. Furthermore, the similarities with of Asian, European, and American haplotypes ranged from 98.09% to 98.81% (Supplementary Table S3). At the same time, full-length sequences from H1 and M1-M3 were 99.24–99.26%% identical, and the similarities with of Asian, European, and American haplotypes ranged from 98.68% to 99.10% (Supplementary Table S4). However, there were one to several single-nucleotide polymorphisms (SNPs) in the full-length mitogenome sequences of all four different samples. The annotated sequences were deposited in National Center for Biotechnology Information (NCBI) GenBank under accession numbers PV649818–PV649821. These four mitogenomes are 13,738 bp in size and include 12 protein-coding (CDS) genes, 22 transfer RNA (tRNA) genes, and 2 ribosomal RNA genes, all of which are well conserved.

### Haplotype network analysis of concatenated three sequences of *cob*, *nad2*, and *cox1*

**Fig. 3:**
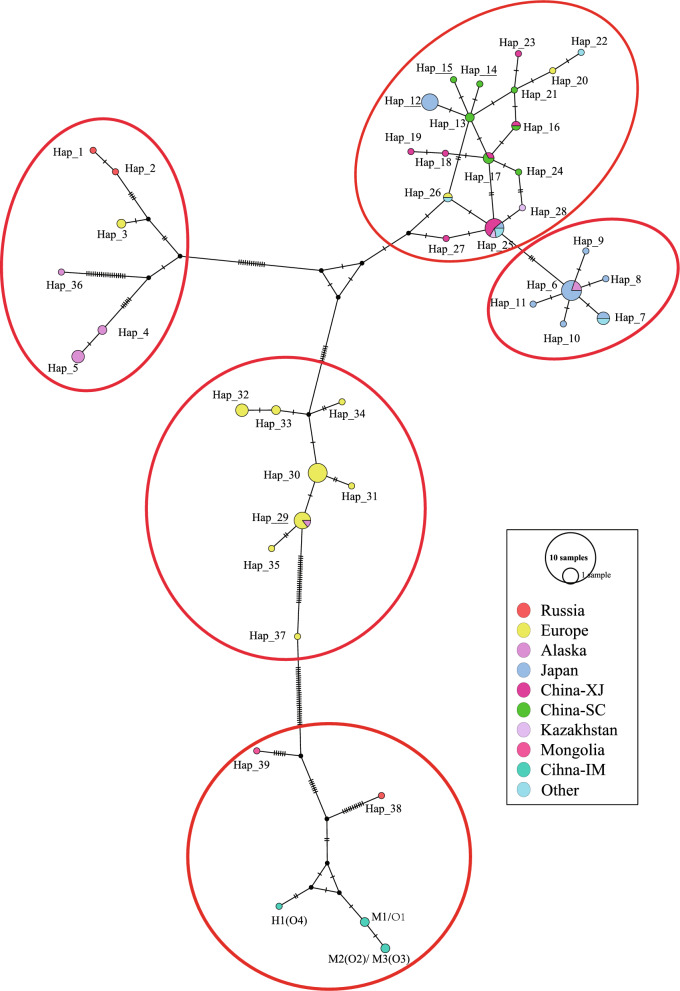
Visualization of networks for the concatenated sequences of the mitochondrial genes *cob*, *nad2*, and *cox1* of *E. multilocularis* from Europe, Russia, China, Japan, and North America, based on the median-joining method. The sizes of the circles correspond to the number of samples representing each sequence, while the colors indicate geographical origins. Dashes on the lines represent mutations. China-XJ: China-Xinjiang; China-SC: China-Sichuan; China-IM: Inner Mongolia, China

**Fig. 4:**
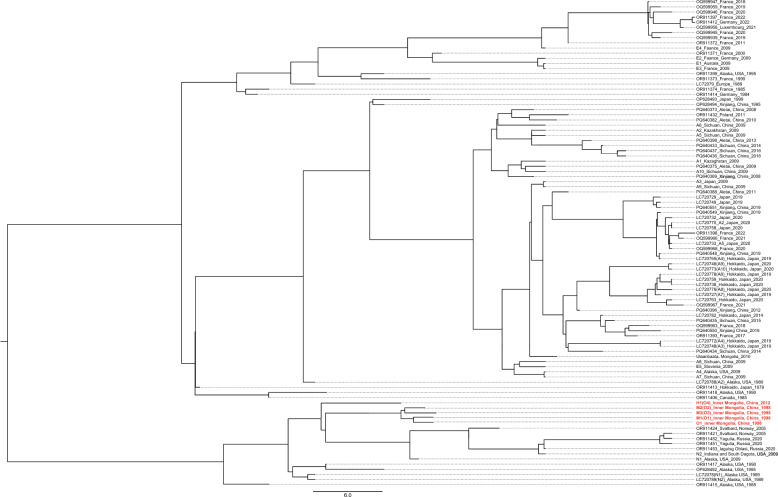
Phylogenetic tree obtained from Bayesian skyline analyses of the concatenated sequences of the mitochondrial genes *cob*, *nad2*, and *cox1* of *E. multilocularis* from Europe, Russia, China, Japan, and North America

Network analysis of the mitogenome sequences recovered eight regions of *E. multilocularis*: China-Inner Mongolia (China-IM), China-Xinjiang (China-XJ), China-Sichuan (China-SC), Mongolia, Japan, Russia, Europe, and Kazakhstan (Fig. 3). All 98 concatenated three-gene sequences of *cob*, *nad2*, and *cox1* were classified into 42 haplotypes, with the different SNPs forming a star representing each haplotype. All 42 haplotypes basically divided into five groups according to geographical distribution (Fig. 4). Haplotypes 41 and 42 from Inner Mongolia, China could be assigned to a distinct haplogroup owing to their high variability. The F_ST_ values between different highly endemic areas were significantly different in other combinations (except for Russia and North America), indicating that *E. multilocularis* populations are highly genetically differentiated in northeast Asia and Alaska, USA (Table 2). However, the F_ST_ values between Inner Mongolia, China and other endemic areas were moderately or significantly different in other combinations, except for Russia and North America.

**Table 2:** Pairwise fixation index (F_ST_) among the concatenated sequences of the genes *cob*, *nad2*, and *cox1* of *E. multilocularis* subpopulations from Russia, Mongolia, Japan, Kazakhstan, North America (Canada and Alaska, Indiana, and South Dakota, USA), Europe (Poland, Germany, Austria, France, Norway, and Slovakia), and CHN-XJ (China: Xinjiang), CHN-SC (China: Sichuan), and CHN-IM (China: Inner Mongolia)

Location	Russia	Mongolia	Japan	Kazakhstan	North America	Europe	China-XJ	China-SC	China-IM
Russia	0.00000								
Mongolia	− 0.05691	0.00000							
Japan	0.75229	0.91540	0.00000						
Kazakhstan	0.24786	0.97590	0.20684	0.00000					
North America	**0.25460**	0.62145	0.48588	0.16782	0.00000				
Europe	0.65306	0.81751	0.64093	0.51296	0.46775	0.00000			
China-XJ	0.68297	0.93392	0.23265	− 0.02253	0.40346	0.59877	0.00000		
China-SC	0.69269	0.94831	0.39574	0.44794	0.44309	0.61542	0.21873	0.00000	
China-IM	**0.63064**	0.86905	0.94297	0.96646	**0.78572**	0.87703	0.95521	0.96260	0.00000

F_ST_ values nearing 1 indicate extreme genetic differentiation between two subpopulations. Significant values are indicated by an asterisk (*P* < 0.05)

The distance-based NJ phylogenetic map clearly shows THAT the concatenated haplotypes of the three sequences were divided into clades representing Europe, Asia, North America, and Inner Mongolia, China (Supplementary Fig. S1). Interestingly, OR911451 and OR911452 from Siberia, Russia, and N1 coexisted in a subclade, while the two sequences of OR911453 and those from Mongolia were grouped into the same clade. Additionally, several sequences from Mongolia and Inner Mongolia were located on the same branch. In this phylogram, the four different geographic clades were all supported by high bootstrap values. These haplotypes from Inner Mongolia, China: H1, O1, and M1–M3 were deeply branched; however, the bootstrap value for their node was not particularly high. The maximum pairwise divergence reached 0.43% when compared with the haplotypes O1 and OR911453.

Rooted branching maps were inferred using maximum-likelihood (ML) and Bayesian methods (Fig. 4). Both approaches produced trees with similar topologies, illustrating distinct geographical branches. The species and lineage delimitation analysis, conducted using four concatenated mitogenome DNA sequences, identified four mitogenome branches. The four sequences from Inner Mongolia were relatively close to lineage sequences found near the Arctic Circle, including those from Siberia, Norway, Alaska, and Canada. The Bayesian phylogenies based on the concatenated sequences of *cob*, *nad2*, and *cox1* from Inner Mongolia, China, and Mongolia indicated that the five sequences clustered into four distinct subclades. Among these, M2, M3, and H1 formed one branch, while M1 and O1 constituted an independent branch. M1 was closely related to the branches of M2, M3, and H1, yet separated from the sequences originating from Mongolia.

### Haplotype network analysis of full-length mitogenome sequences

**Fig. 5:**
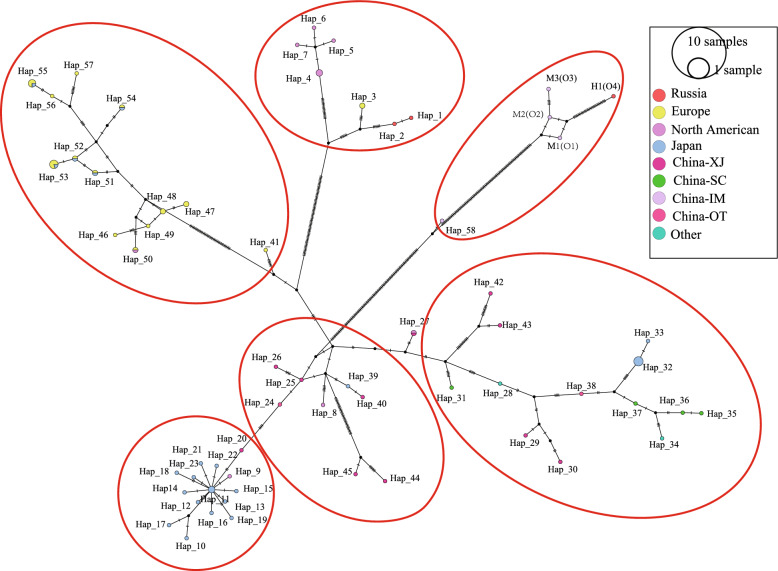
Visualization of networks for the full-length mitogenome sequences of *E. multilocularis* from Europe, Russia, China, Japan, and North America, based on the median-joining method. The sizes of the circles correspond to the number of samples representing each sequence, while the colors indicate their geographical origins. Dashes on the lines represent mutations. China-XJ: China-Xinjiang; China-SC: China-Sichuan; China-IM: Inner Mongolia, China; China-OT: China-Other

**Fig. 6:**
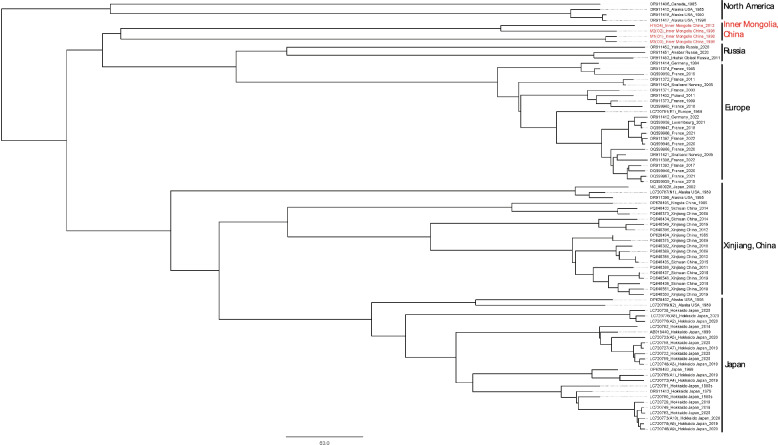
Phylogenetic tree derived from Bayesian skyline analyses of full-length mitogenome sequences of *E. multilocularis* from Europe, Russia, China, Japan, and North America, constructed using the median-joining method

Network analysis of the full-length mitogenome sequences identified eight regions of *E. multilocularis*: China-IM, China-XJ, China-SC, other regions of China, North America, Japan, Russia, and Europe (Fig. 5). A total of 79 sequences were classified into 62 haplotypes, which were grouped into six categories primarily based on geographical distribution (Fig. 6). Haplotype 59–62 from Inner Mongolia, China, was assigned to a distinct haplogroup owing to its high variability. Consistent with these findings, the elevated F_ST_ values between endemic regions and significant differences in all combinations (except between Russia and North America) indicate a high level of genetic differentiation among *E. multilocularis* populations in northeast Asia and Alaska, USA (Table 3). However, moderate to significant differences in F_ST_ values were observed between Inner Mongolia, China and other endemic regions in all combinations, except for Russia and North America.

**Table 3:** Pairwise fixation index (F_ST_) among the full sequences of *E. multilocularis* subpopulations from Russia, Japan, North America (Canada and Alaska, Indiana, and South Dakota, USA), Europe (Poland, Germany, Austria, France, Norway, and Slovakia), and CHN-XJ (China: Xinjiang), CHN-SC (China: Sichuan), and CHN-IM (China: Inner Mongolia)

Location	Russia	Japan	North America	Europe	China-XJ	China-SC	China-IM
Russia	0.00000						
Japan	0.78305	0.00000					
North America	**0.22084**	0.72718	0.00000				
Europe	0.63005	0.62091	0.60624	0.00000			
China-XJ	0.60806	0.17289	0.58463	0.53034	0.00000		
China-SC	0.57203	0.94831	0.39574	0.44794	0.44309	0.00000	
China-IM	**0.44220**	0.90590	**0.75922**	0.82263	0.82692	0.85587	0.00000

F_ST_ values nearing 1 indicate extreme genetic differentiation between two subpopulations. Significant values are indicated by an asterisk (*P* < 0.05)

Similar to the results of the three concatenated sequences mentioned above, the distance-based NJ phylogram clearly demonstrated that the haplotypes of the concatenated sequences were divided into clades representing Europe, Asia, North America, and Inner Mongolia, China (Supplementary Fig. S2). Interestingly, the OR911451 and OR911452 haplotypes from Siberia, Russia coexisted with the North American haplotypes N1 and N2 within the same clade. The sequences of OR911453 and other sequences from Inner Mongolia were situated within the same clade. The sequences from Inner Mongolia, China, specifically H1, and M1–M3, exhibited significant branching, with a high bootstrap value for their node. The maximum pairwise divergence between the sequences from Inner Mongolia and OR911453 ranged from 0.64% to 1.29%.

Rooted branching plots were inferred using maximum-likelihood (ML) and Bayesian methods (Fig. 6). Both methods produced trees with similar topologies, revealing four distinct geographical clades. In terms of geographical distribution, these trees appeared monophyletic; however, the branching nodes separating different clades were not strongly supported by Bayesian statistics. The species and lineage delimitation analysis, conducted using four full-length sequences, identified four distinct sequence branches. The sequences from Inner Mongolia were relatively close to those from Siberia, Russia. Among these, M2 and H1 formed one branch, while M1 and M3 constituted an independent branch. All four sequences from Inner Mongolia, China were closely related to the sequence branches from Alaska and Russia. Notably, the four full-length sequences were exclusive to Inner Mongolia China, and have not been previously reported elsewhere in the world. Bayesian phylogeny revealed three clearly distinguished groups based on branching patterns.

## Discussion

There have been several reports on *E. multilocularis* from Siberia since the 1950s [9, 12, 13, 35]. However, these early studies primarily focused on classifying *E. sibiricensis* on the basis of epidemiological and morphological characteristics, with limited attention given to *E. multilocularis* in the region at that time [6, 12, 35]. This lack of data led some researchers to make inappropriate classifications. Subsequently, Nakao conducted a comprehensive analysis of sequences from China, Japan, Kazakhstan, Europe, and North America, focusing on three gene segments. These findings formed the foundation for much of the subsequent research in this field. Nevertheless, recent advancements in molecular biology have enabled the analysis of the full-length mitogenome of *E. multilocularis*, revealing certain limitations in Nakao’s classification system [21, 24, 36].

The primary shortcomings include: (1) the limited number of mitogenome bases analyzed, and (2) insufficient accuracy in classification, which does not align with the results obtained from full-length sequence typing [21]. On the basis of these considerations, we sequenced the full-length mitogenome of samples from Inner Mongolia, China, reviewed previous research findings, and concluded that the Inner Mongolia haplotype corresponds to the *E. m. sibiricensis* reported in earlier studies [6]. In Academician Tang’s analysis, the three sequences were not sequenced owing to the limitations of the available conditions at that time. The three sequences were classified solely on the basis of morphological and pathological changes: sequence I belonged to *E. m. sibiricensis*, sequence II belonged to *E. m. multilocularis*, and sequence III was not clearly defined [12]. The sequences M1, M2, and M3 analyzed in our study correspond to I, II, and III in Academician Tang’s study, along with one patient-derived sequence from Hulunbuir, Inner Mongolia, which provided full-length sequence data for all four samples. In the two Bayesian analyses, all four sequences were located on the same branch, and their sequence similarity differed significantly from the Asian, European, and North American haplotypes.

Our phylogenetic analyses indicate a clear geographical separation between four sequences from Inner Mongolia and other sequence clusters. Moreover, the mitogenome phylogeny and PopArt analysis revealed a geographical separation into six clusters (Fig. 3). A geographic pattern of genetic differentiation was observed in Northeast Asia (including Inner Mongolia, China, Mongolia, and Siberia, Russia), with high sequence similarity associated with specific geographical regions. These findings are consistent with previous observations, based on mitogenome sequences, that the *E. multilocularis* haplotype complex exhibits a high degree of lineage diversity across its range [6]. Interestingly, the new clade of four mitogenomes that we detected represents haplotype O and was found exclusively in Inner Mongolia, China. This clade is distinctly separate from other clade, suggesting that these species has been isolated from other populations for some time. The unique ecosystem of the Hulunbuir steppe may have allowed a different population to evolve there [12, 35].

We observed discrepancies in the topologies presented in Figs. 4 and 6. The primary cause of these discrepancies lies in the inconsistency between the similarities of the concatenated complete sequences of *cob*, *nad2*, and *cox1* and the full-length sequences. As illustrated in Supplementary Tables 3 and 4, certain variations exist in the similarity values among specific sequences when compared with the full-length sequences. We also found certain discrepancies among some samples in different clusters, which can be attributed to the same reasons related to topology as discussed above. The French samples fall within the Asian clade. These findings are consistent with those reported by previous studies [19]. For instance, haplotype A2 is widely distributed across Asia, Europe, and the Americas. Given that the complete sequence comprises 12 full coding sequences, the overall classification of haplotypes may be considered to be more reliable. In contrast, the classification using concatenated complete sequences of the mitochondrial genes *cob*, *nad2*, and *cox1* was less rigorous owing to technical limitations at the time. For example, the initial classification was proposed by Nakao et al., and later, Hayashi et al. compared the complete sequence with the concatenated three-gene sequence and identified discrepancies between the two classifications [6, 19]. In this study, haplotype classification primarily relies on the complete sequence, supplemented by the classification criteria established by Nakao et al. [6].

The sequences of sibling species/lineages from Mongolia and Siberia have also been detected in other regions of Northeast Asia with high similarity. The similarity to the Inner Mongolia sequence may be attributed to differences in hosts, closer geographical proximity, and distinct ecological environments. Geographically, Inner Mongolia, China, Mongolia, Siberia, Japan, North America, and even the Arctic Circle, including Norway and Greenland, are relatively close [37]. We found N2 sequences present in the Far East of Russia as well as a small number of N1 and N2 sequences in Hokkaido, Japan [21]. Integrating geographical location data with genetic analyses helps explain the close genetic distance between Inner Mongolia, Russia, and North America. Several lines of evidence suggest that human-mediated translocation of infected animals plays an important role in the distribution of *E. multilocularis* in the intermediate host *Myodes rutilus*, which constitutes approximately 50% of the population on the islands [21]. During 1916–1917, 15 pairs of blue foxes (*Vulpes lagopus*) were introduced to the Middle Kuril Islands for the fox fur industry [38]. Subsequently, infected foxes were transferred to other Kuril Islands [39, 40], and foxes from the Kuril Islands have been identified as sources of parasites on Rebun Island and the main island of Hokkaido.

Since *E. sibiricensis* was also once found on St Lawrence Island, there are certain limitations to our study. Therefore, more isolates from Siberian, northwest China, Germany, and even the Arctic Circle endemic areas are needed to elucidate the genetic and evolutionary history of *E. multilocularis* in order to demonstrate that *E. sibiricensis* is the O haplotype. Specifically, we considered the phylogenetic analyses of haplotypes O1–O4, N1, and N2 in terms of spatial and ecological separation. Incomplete families of *E. multilocularis* can be addressed using the obtained genetic data.

## Conclusion

In conclusion, we classified the four sequences from Inner Mongolia, China, into four haplotypes: O1–O4. We believe that *E. sibiricensis* represents the O1 haplotype and is widely distributed in northeast China, Siberia, and regions near the Arctic Circle. We speculate that northeast Asia and its adjacent regions are critical areas in the evolution of the O haplotype. The results of this study, particularly for samples from Northeast Asia, supplement Nakao’s previous analysis and confirm for the first time that the samples collected by Academician Tang and the *E. m. sibiricensis* reported by Rausch and Schiller belong to haplotypes originating from Inner Mongolia, China. This finding is of great significance for understanding the genetic and evolutionary history of *E. multilocularis*. This study has inherent limitations that should be acknowledged. First, it utilizes a small sample size, consisting of only four *E. multilocularis* isolates exclusively from Inner Mongolia, China, and lacks isolates from key regions such as Siberia, northwest China, and the Arctic Circle—this restricts the generalizability of its conclusions regarding the global distribution and evolutionary context of the O1 haplotype. Second, the analysis depends solely on mitochondrial genome data, with no integration of nuclear genetic markers (e.g., microsatellites or whole-genome sequences); as a result, it cannot fully capture the comprehensive population structure and evolutionary history of *E. multilocularis*, nor can it precisely determine the timing of geographic isolation for the O haplotype.

## Supplementary Information

Supplementary Material 1: Fig. S1. Phylogenetic tree of the concatenated sequences of the mitogenome genes *cob*, *nad2*, and *cox1* from* Echinococcus multilocularis* haplotypes collected in Inner Mongolia, China (designated as O), along with published *Echinococcus multilocularis* haplotypes from Europe (E1–E5), Asia (A1–A10), and North America (N1 and N2). These sequences were originally published by Nakao et al. (2009) and Guo et al. (2025). The scale bar represents the estimated number of substitutions per site. The phylogenetic tree was validated using 1000 bootstrap replicates. The haplotypes reported in this paper, as well as the O1 haplotypes, are indicated in red.

Supplementary Material 2: Fig. S2. Phylogenetic tree of the full-length mitogenomes of *Echinococcus multilocularis* haplotypes from Inner Mongolia, China (O), alongside published *Echinococcus multilocularis* haplotypes from Europe, Russia, China, Japan, and North America. The scale bar represents the estimated number of substitutions per site. This phylogenetic tree was also validated using 1000 bootstrap replicates. The four haplotypes reported in this paper are highlighted in red.

Supplementary Material 3 : Table S1. Accession numbers for the concatenated sequences of the mitogenome genes *cob*, *nad2*, and *cox1* of *E. multilocularis* cited for haplotype 1–42 from GenBank in this study.

Supplementary Material 4: Table S2. Accession numbers for the full mitogenome sequences of *E. multilocularis* cited for haplotypes 1–62 from GenBank in this study.

Supplementary Material 5: Table S3. Similarity analysis (%) of concatenated sequences of *cob*, *nad2*, and *cox1* of *E. multilocularis* in northeast Asia (including Inner Mongolia, China, Mongolia, and Siberia, Russia) compared with other haplotypes published by Nakao et al. [6].

Supplementary Material 6: Table S4. Similarity analysis (%) of full-length mitogenome sequences of *E. multilocularis* in northeast Asia (including Inner Mongolia, China, and Siberia, Russia) compared with other haplotypes published by Hayashi et al. [21].

## Data Availability

Data supporting the main conclusions of this study are included in the manuscript.

## Abbreviations

AE: Alveolar echinococcosis
E: Europe
A: Asian
N: North American
O: Inner Mongolia
AL: Adventitial layer
LL: Laminated layers
GL: Germinal layer
IHC: Immunohistochemical
H&E: Hematoxylin and eosin
α-SMA: α-Smooth muscle actin
Pv: Parasitic vesicle
CT: Computed tomography
PSCs: Protoscoleces
F_ST_: Pairwise fixation index
NJ: Neighbor-joining
GTR: Generalized time-reversible
