# Supplementary material for: Analysis of four Echinococcus multilocularis mitogenome sequences from Inner Mongolia, China: supporting the hypothesis that E. sibiricensis is confirmed as the O1 haplotype
Source: Parasit Vectors. 2025 Nov 4;18:444. doi: 10.1186/s13071-025-07057-7 (PMC12584506; doi:10.1186/s13071-025-07057-7)
Supplement: Supplementary file 3 — Supplementary Material 3 : Table S1. Accession numbers for the concatenated sequences of the mitogenome genes cob, nad2, and cox1 of E. multilocularis cited for haplotype 1–42 from GenBank in this study. [file 13071_2025_7057_MOESM3_ESM.docx]

**Supplementary Table S1. Accession numbers for the** **concatenated sequences of the mitochondrial genes *cob*, *nad2*, and *cox1* of *E. multilocularis* cited for haplotype 1 - haplotype 42 from GenBank in this study**

| **Haplotype** | **Genotype ^1^** | **Genotype ^2^** | **Accession Number** | **Location** | **Time** |
| --- | --- | --- | --- | --- | --- |
| Hap1 |  |  | OR911451 | Yakutia, Russia | ND |
| Hap2 |  |  | OR911452 | Yakutia, Russia | ND |
| Hap3 |  |  | OR911453 | Irkutsk, Russia | 2010-2012 |
| Hap4 |  |  | AB510026+AB461411+AB510025 | Ulaanbaatar, Mongolia | 2009 |
| Hap5 | A4 | A1  A16  A1  A5  A7  A8  A9  A10  A1 | OR911413  LC720738  LC720782  LC720765  LC720733  LC720727  LC720776  LC720746  LC720773  LC720786  OR911406  PQ640398 | Japan  Japan  Japan  Japan  Japan  Japan  Japan  Japan  Japan  Alaska, USA  Canada  Xinjiang, China | 1979  2020  2014  2019  2020  2019  2020  2020  2020  2009  1985  2013 |
| Hap6 | A3 |  | AB461399 + AB461407 + AB461416  LC72078 | Japan  Japan | 2009  1980s |
| Hap7 |  | A2  A2  A2  A3 | LC720729  LC720732  LC720749  LC720758  LC720759  LC720770  LC720748 | Japan  Japan  Japan  Japan  Japan  Japan  Japan | 2019  2020  2019  2020  2020  2020  2019 |
| Hap8 |  |  | LC720763 | Japan | 2020 |
| Hap9 |  |  | LC720780 | Japan | 1980s |
| Hap10 | A2 | A17 | OP628493  AB461398 + AB461406 + AB461416  LC720788  OR911418  PQ640396  PQ640388  PQ640382  PQ640373  OP628494 | Japan  Kazakhstan  Alaska, USA  Alaska, USA  Xinjiang, China  Xinjiang, China  Xinjiang, China  Xinjiang, China  Xinjiang, China | 1999  2009  1989  1990  2012  2011  2010  2008  1995 |
| Hap11 | A4 |  | LC720772 | Japan | 2019 |
| Hap12 | A6 |  | LC720778 | Japan | 2019 |
| Hap13 | A1 |  | AB461398+AB461406+AB461415 | Kazakhstan | 2009 |
| Hap14 | E2 |  | OR911399  AB461395 + AB461403 + AB461414  OR911372  OR911374  OR911412  OR911414  OQ599958 | Alaska, USA  France  France  France  Germany  Germany  Luxembourg | 1995  2009  2011  1985  2022  1984  2021 |
| Hap15 |  |  | OR911417  OR911415 | Alaska, USA  Alaska, USA | 1990  1989 |
| Hap16 | N1 | N2 | LC720789  LC720787  AB461401 + AB461409 + AB461418  OP628492 | Alaska, USA  Alaska, USA  Alaska, USA  Alaska, USA | 1989  1989  2009  ND |
| Hap17 | N2 |  | AB461400 + AB461410 + AB461419 | Indiana, USA | 2009 |
| Hap18 | E1 |  | AB461395 + AB461403 + AB461412 | Austria | 2009 |
| Hap19 | E3 |  | AB461395 + AB461404 + AB461413 | France | 2009 |
| Hap20 | E4 |  | AB461396 + AB461404 + AB461414  OR911371  OR911373  OR911397  OQ599946  OQ599939  OQ599947  OQ599959  OQ599945 | France  France  France  France  France  France  France  France  France | 2009  2000  1999  2022  2020  2019  2018  2019  2020 |
| Hap21 | E5 |  | AB461397 + AB461405 + AB461414  OR911398  OQ599966  OQ599967 | Slovenia  France  France  France | 2009  2022  2021  2021 |
| Hap22 |  |  | OQ599968 | France | 2020 |
| Hap23 |  | A12 | LC72079 | Europe | 1951 |
| Hap24 |  |  | OR911421  OR911424 | Norway  Norway | 2005  2005 |
| Hap25 |  |  | OR911393 | France | 2017 |
| Hap26 |  | A10 | OR911432  AB461398+AB461406 + AB477011 | Poland  Sichuan, China | 2011 |
| Hap27 |  |  | OQ599963 | France | 2018 |
| Hap28 |  | A5 | AB461398 + AB461408 + AB477011  PQ640433 | Sichuan, China  Sichuan, China | 2014 |
| Hap29 |  |  | PQ640434  PQ640551 | Sichuan, China  Xinjiang, China | 2014  2019 |
| Hap30 |  |  | PQ640435 | Sichuan, China | 2015 |
| Hap31 |  |  | PQ640436  PQ640437  PQ640369 | Sichuan, China  Sichuan, China  Xinjiang, China | 2016  2016  2008 |
| Hap32 | A6 |  | AB461398 + AB461408 + AB477011 | Sichuan, China |  |
| Hap33 | A7 |  | AB461398 + AB461408 + AB477012 | Sichuan, China |  |
| Hap34 | A8 |  | AB477009 + AB461408 + AB477010 | Sichuan, China |  |
| Hap35 | A9 |  | AB461398 + AB461408 + AB477017 | Sichuan, China |  |
| Hap36 |  |  | PQ640375 | Xinjiang, China | 2009 |
| Hap37 |  |  | PQ640548 | Xinjiang, China | 2019 |
| Hap38 |  |  | PQ640549 | Xinjiang, China | 2019 |
| Hap39 |  |  | PQ640550 | Xinjiang, China | 2019 |
| Hap40 | O1 |  | AB461411+AB461402+ AB461420  M1 | Inner Mongolia, China  Inner Mongolia, China | 1998^*^  1998^*^ |
| Hap41 |  |  | M2  M3 | Inner Mongolia, China  Inner Mongolia, China | 1998^*^  1998^*^ |
| Hap42 |  |  | H1 | Inner Mongolia, China | 2012 |

^1, 2^ Identified genotype; ^a^ Complete sequence; ^b^ Concatenated three sequences of *cob*, *nad2* and *cox1* of *E. multilocularis* from full sequences; ND, unknown; ^*^ Maintained in gerbils since 1998
