# Supplementary material for: Analysis of four Echinococcus multilocularis mitogenome sequences from Inner Mongolia, China: supporting the hypothesis that E. sibiricensis is confirmed as the O1 haplotype
Source: Parasit Vectors. 2025 Nov 4;18:444. doi: 10.1186/s13071-025-07057-7 (PMC12584506; doi:10.1186/s13071-025-07057-7)
Supplement: Supplementary file 4 — Supplementary Material 4: Table S2. Accession numbers for the full mitogenome sequences of E. multilocularis cited for haplotypes 1–62 from GenBank in this study. [file 13071_2025_7057_MOESM4_ESM.docx]

**Supplementary Table S2.** **Accession numbers for the full mitogenome sequences of *E. multilocularis* cited for haplotypes haplotype 1 - haplotype 62 from GenBank in this study**

| **Haplotype** | **Genotype ^a^** | **Accession Number** | **Location** | **Time** |
| --- | --- | --- | --- | --- |
| Hap1 |  | OR911451 | Yakutia, Russia | ND |
| Hap2 |  | OR911452 | Yakutia, Russia | ND |
| Hap3 |  | OR911421  OR911424 | Norway  Norway | 2005  2005 |
| Hap4 |  | OR911399  OR911417  OR911415 | Alaska, USA  Alaska, USA  Alaska, USA | 1995  1990  1985 |
| Hap5 |  | OP62849 | Alaska, USA | 1985 |
| Hap6 | N2 | LC720789() | Alaska, USA | 1989 |
| Hap7 | N1 | LC720787 | LC720787 | 1989 |
| Hap8 |  | OR911418 | Alaska, USA | 1990 |
| Hap9 | A5 | OR911406 | Canada | 1985 |
| Hap10 | A8 | AB018440 | Japan | 1999 |
| Hap11 | A1 | LC720738  OR911413  LC720765 | Japan  Japan  Japan | 2020  1979  2019 |
| Hap12 |  | LC720780 | Japan | 1980s |
| Hap13 | A5 | LC720733 | Japan | 2020 |
| Hap14 | A8 | LC720776 | Japan | 2020 |
| Hap15 | A9 | LC720746 | Japan | 2020 |
| Hap16 | A10 | LC720773 | Japan | 2020 |
| Hap17 |  | LC720781 | Japan | 1980s |
| Hap18 |  | LC720782 | Japan | 2014 |
| Hap19 | A4 | LC720772 | Japan | 2019 |
| Hap20 |  | PQ640398 | Xinjiang, China | 2013 |
| Hap21 |  | LC720763 | Japan | 2020 |
| Hap22 | A6 | LC720778 | Japan | 2019 |
| Hap23 | A7 | LC720727 | Japan | 2019 |
| Hap24 |  | PQ640396 | China | 2012 |
| Hap25 |  | PQ640382 | Xinjiang, China | 2010 |
| Hap26 |  | PQ640388 | Xinjiang, China | 2011 |
| Hap27 |  | PQ640369 | Xinjiang, China | 2008 |
| Hap28 |  | PQ640436 | Sichuan, China | 2016 |
| Hap29 |  | PQ640548 | Xinjiang, China | 2019 |
| Hap30 |  | PQ640550 | Xinjiang, China | 2019 |
| Hap31 |  | PQ640437 | Sichuan, China | 2016 |
| Hap32 | A2 | LC720729  LC720732  LC720749  LC720758  LC720759  LC720770 | Japan  Japan  Japan  Japan  Japan  Japan | 2019  2020  2019  2020  2020  2020 |
| Hap333 | A3 | LC720748 | Japan | 2019 |
| Hap34 |  | OQ599968 | France | 2020 |
| Hap35 |  | PQ640434 | Sichuan, China | 2014 |
| Hap36 |  | PQ640435 | Sichuan, China | 2015 |
| Hap37 |  | PQ640433 | Sichuan, China | 2014 |
| Hap38 |  | OP628495 | Ningxia, China | 1995 |
| Hap39 |  | OP628493 | Japan | 1999 |
| Hap40 |  | OP628494 | Xinjiang, China | 1995 |
| Hap41 |  | OR911432 | Poland | 2011 |
| Hap42 |  | PQ640549 | Xinjiang, China | 2019 |
| Hap43 |  | PQ640551 | Xinjiang, China | 2019 |
| Hap44 |  | PQ640375 | Xinjiang, China | 2009 |
| Hap45 |  | PQ640373 | Xinjiang, China | 2008 |
| Hap46 | E1 | LC720791 | Europe | 1989 |
| Hap47 |  | OR911372  OR911374 | France  France | 2011  1985 |
| Hap48 |  | OR911412  OR911414 | Germany  Germany | 2022  1984 |
| Hap49 |  | OQ599958 | Luxembourg | 2021 |
| Hap50 |  | OQ599959 | France | 2019 |
| Hap51 |  | OR911371 | France | 2000 |
| Hap52 |  | OR911373 | France | 1999 |
| Hap53 |  | OQ599946  OQ599945  OQ599947  OQ599939 | France  France  France  France | 2020  2020  2018  2019 |
| Hap54 |  | OR911397 | France | 2022 |
| Hap55 |  | OR911398  OQ599966  OQ599967 | France  France  France | 2022  2021  2021 |
| Hap56 |  | OQ599963 | France | 2018 |
| Hap57 |  | OR911393 | France | 2017 |
| Hap58 |  | OR911453 | Irkutsk, Russia | 2010-2012 |
| Hap59 |  | M1 | Inner Mongolia, China | 1998^*^ |
| Hap60 |  | M2 | Inner Mongolia, China | 1998^*^ |
| Hap61 |  | M3 | Inner Mongolia, China | 1998^*^ |
| Hap62 |  | H1 | Inner Mongolia, China | 2012 |

**^a^** Identified genotype; ND, unknown; ^*^ Maintained in gerbils since 1998
