# Supplementary material for: Analysis of four Echinococcus multilocularis mitogenome sequences from Inner Mongolia, China: supporting the hypothesis that E. sibiricensis is confirmed as the O1 haplotype
Source: Parasit Vectors. 2025 Nov 4;18:444. doi: 10.1186/s13071-025-07057-7 (PMC12584506; doi:10.1186/s13071-025-07057-7)
Supplement: Supplementary file 5 — Supplementary Material 5: Table S3. Similarity analysis (%) of concatenated sequences of cob, nad2, and cox1 of E. multilocularis in northeast Asia (including Inner Mongolia, China, Mongolia, and Siberia, Russia) compared with other haplotypes published by Nakao et al. [6]. [file 13071_2025_7057_MOESM5_ESM.docx]

**Supplementary Table S3. Similarity analysis (%) of concatenated sequences of *cob*, *nad2*, and *cox1* of *E. multilocularis* in Northeast Asia (including Inner Mongolia, China, Mongolia, and Siberia, Russia) compared to other haplotypes published by Nakao et al. [6]**

|  | **H1** | **O1** | **M1** | **M2** | **M3** | **Mongolia** | **OR911453** | **OR911452** | **OR911451** | N1 | N2 | E1 | E2 | E3 | E4 | E5 | A1 | A2 | A3 | A4 | A5 | A6 | A7 | A8 | A9 | A10 |
| --- | --- | --- | --- | --- | --- | --- | --- | --- | --- | --- | --- | --- | --- | --- | --- | --- | --- | --- | --- | --- | --- | --- | --- | --- | --- | --- |
| H1 | 100 |  |  |  |  |  |  |  |  |  |  |  |  |  |  |  |  |  |  |  |  |  |  |  |  |  |
| O1 | 99.89 | 100 |  |  |  |  |  |  |  |  |  |  |  |  |  |  |  |  |  |  |  |  |  |  |  |  |
| M1 | 99.89 | 100 | 100 |  |  |  |  |  |  |  |  |  |  |  |  |  |  |  |  |  |  |  |  |  |  |  |
| M2 | 99.86 | 99.97 | 99.97 | 100 |  |  |  |  |  |  |  |  |  |  |  |  |  |  |  |  |  |  |  |  |  |  |
| M3 | 99.86 | 99.97 | 99.97 | 100 | 100 |  |  |  |  |  |  |  |  |  |  |  |  |  |  |  |  |  |  |  |  |  |
| Mongolia | 99.49 | 99.52 | 99.52 | 99.55 | 99.55 | 100 |  |  |  |  |  |  |  |  |  |  |  |  |  |  |  |  |  |  |  |  |
| OR911453 | 99.54 | 99.57 | 99.57 | 99.54 | 99.54 | 99.31 | 100 |  |  |  |  |  |  |  |  |  |  |  |  |  |  |  |  |  |  |  |
| OR911451 | 98.12 | 98.15 | 98.15 | 98.18 | 98.18 | 98.55 | 98.11 | 100 |  |  |  |  |  |  |  |  |  |  |  |  |  |  |  |  |  |  |
| OR911452 | 98.12 | 98.15 | 98.15 | 98.18 | 98.18 | 98.55 | 98.11 | 100 | 100 |  |  |  |  |  |  |  |  |  |  |  |  |  |  |  |  |  |
| N1 | 98.09 | 98.12 | 98.12 | 98.15 | 98.15 | 98.52 | 98.08 | 98.11 | 100 | 100 |  |  |  |  |  |  |  |  |  |  |  |  |  |  |  |  |
| N2 | 98.15 | 98.18 | 98.18 | 98.21 | 98.21 | 98.52 | 98.08 | 98.11 | 99.63 | 99.34 | 100 |  |  |  |  |  |  |  |  |  |  |  |  |  |  |  |
| E1 | 98.81 | 98.85 | 98.85 | 98.88 | 98.88 | 98.99 | 98.69 | 98.08 | 99.22 | 99.22 | 98.72 | 100 |  |  |  |  |  |  |  |  |  |  |  |  |  |  |
| E2 | 98.11 | 98.15 | 98.15 | 98.18 | 98.18 | 98.60 | 98.05 | 98.08 | 98.75 | 98.75 | 99.11 | 99.34 | 100 |  |  |  |  |  |  |  |  |  |  |  |  |  |
| E3 | 98.08 | 98.12 | 98.12 | 98.15 | 98.15 | 98.57 | 98.02 | 98.69 | 99.08 | 99.08 | 99.08 | 99.25 | 99.92 | 100 |  |  |  |  |  |  |  |  |  |  |  |  |
| E4 | 98.15 | 98.18 | 98.18 | 98.21 | 98.21 | 98.63 | 98.08 | 98.05 | 99.05 | 99.05 | 99.14 | 99.31 | 99.97 | 99.94 | 100 |  |  |  |  |  |  |  |  |  |  |  |
| E5 | 98.11 | 98.15 | 98.15 | 98.18 | 98.18 | 98.60 | 98.05 | 98.02 | 99.11 | 99.11 | 99.11 | 99.22 | 99.89 | 99.86 | 99.92 | 100 |  |  |  |  |  |  |  |  |  |  |
| A1 | 98.24 | 98.27 | 98.27 | 98.30 | 98.30 | 98.78 | 98.17 | 98.08 | 99.08 | 99.08 | 99.22 | 99.05 | 99.54 | 99.52 | 99.57 | 99.54 | 100 |  |  |  |  |  |  |  |  |  |
| A2 | 98.27 | 98.30 | 98.30 | 98.34 | 98.34 | 98.81 | 98.21 | 98.05 | 99.28 | 99.28 | 99.25 | 99.08 | 99.57 | 99.54 | 99.60 | 99.57 | 99.97 | 100 |  |  |  |  |  |  |  |  |
| A3 | 98.15 | 98.18 | 98.18 | 98.21 | 98.21 | 98.69 | 98.08 | 98.17 | 99.31 | 99.31 | 99.14 | 98.96 | 99.46 | 99.43 | 99.49 | 99.46 | 99.86 | 99.89 | 100 |  |  |  |  |  |  |  |
| A4 | 98.18 | 98.21 | 98.21 | 98.24 | 98.24 | 98.72 | 98.11 | 98.21 | 99.20 | 99.20 | 99.17 | 98.99 | 99.49 | 99.46 | 99.52 | 99.49 | 99.89 | 99.92 | 99.97 | 100 |  |  |  |  |  |  |
| A5 | 98.24 | 98.27 | 98.27 | 98.30 | 98.30 | 98.78 | 98.17 | 98.08 | 99.22 | 99.22 | 99.23 | 99.05 | 99.54 | 99.52 | 99.57 | 99.54 | 99.89 | 99.92 | 99.80 | 99.83 | 100 |  |  |  |  |  |
| A6 | 98.21 | 98.24 | 98.24 | 98.27 | 98.27 | 98.75 | 98.14 | 98.11 | 99.28 | 99.28 | 99.20 | 99.02 | 99.52 | 99.49 | 99.54 | 99.52 | 99.86 | 99.89 | 99.77 | 99.80 | 98.60 | 100 |  |  |  |  |
| A7 | 98.21 | 98.24 | 98.24 | 98.27 | 98.27 | 98.75 | 98.14 | 98.17 | 99.25 | 99.25 | 99.20 | 99.02 | 99.52 | 99.49 | 99.54 | 99.52 | 99.86 | 99.89 | 99.77 | 99.80 | 99.97 | 99.94 | 100 |  |  |  |
| A8 | 98.21 | 98.24 | 98.24 | 98.27 | 98.27 | 98.70 | 98.14 | 98.14 | 99.25 | 99.25 | 99.14 | 98.96 | 99.46 | 99.43 | 99.49 | 99.46 | 99.80 | 99.83 | 99.72 | 99.74 | 99.97 | 99.89 | 99.89 | 100 |  |  |
| A9 | 98.18 | 98.21 | 98.21 | 98.24 | 98.24 | 98.72 | 98.11 | 98.14 | 99.20 | 99.20 | 99.17 | 98.99 | 99.49 | 99.46 | 99.52 | 99.49 | 99.94 | 99.92 | 99.80 | 99.83 | 99.92 | 99.92 | 99.92 | 99.86 | 99.89 |  |
| A10 | 98.30 | 98.33 | 98.33 | 98.37 | 98.37 | 98.84 | 98.24 | 98.14 | 99.22 | 99.22 | 99.28 | 99.11 | 99.60 | 99.57 | 99.63 | 99.60 | 99.94 | 99.97 | 99.86 | 99.89 | 99.94 | 99.92 | 99.92 | 99.86 | 99.89 | 100 |
