# Supplementary material for: Analysis of four Echinococcus multilocularis mitogenome sequences from Inner Mongolia, China: supporting the hypothesis that E. sibiricensis is confirmed as the O1 haplotype
Source: Parasit Vectors. 2025 Nov 4;18:444. doi: 10.1186/s13071-025-07057-7 (PMC12584506; doi:10.1186/s13071-025-07057-7)
Supplement: Supplementary file 6 — Supplementary Material 6: Table S4. Similarity analysis (%) of full-length mitogenome sequences of E. multilocularis in northeast Asia (including Inner Mongolia, China, and Siberia, Russia) compared with other haplotypes published by Hayashi et al. [21]. [file 13071_2025_7057_MOESM6_ESM.docx]

**Supplementary Table S4. Similarity analysis (%) of full-length mitogenome sequences of *E. multilocularis* in Northeast Asia (including Inner Mongolia, China, and Siberia, Russia) compared to other haplotypes published by Hayashi et al. [21]**

|  | H1 | M1 | M2 | M3 | OR911453 | OR911452 | OR911451 | N1 | N2 | E1 | A1 | A2 | A3 | A4 | A5 | A6 | A7 | A8 | A9 | A10 |
| --- | --- | --- | --- | --- | --- | --- | --- | --- | --- | --- | --- | --- | --- | --- | --- | --- | --- | --- | --- | --- |
| H1 | 100 |  |  |  |  |  |  |  |  |  |  |  |  |  |  |  |  |  |  |  |
| M1 | 99.26 | 100 |  |  |  |  |  |  |  |  |  |  |  |  |  |  |  |  |  |  |
| M2 | 99.26 | 99.98 | 100 |  |  |  |  |  |  |  |  |  |  |  |  |  |  |  |  |  |
| M3 | 99.24 | 99.95 | 99.97 | 100 |  |  |  |  |  |  |  |  |  |  |  |  |  |  |  |  |
| OR911453 | 98.71 | 98.25 | 98.25 | 98.23 | 100 |  |  |  |  |  |  |  |  |  |  |  |  |  |  |  |
| OR911452 | 98.71 | 98.25 | 98.25 | 98.23 | 100 | 100 |  |  |  |  |  |  |  |  |  |  |  |  |  |  |
| OR911451 | 99.03 | 99.74 | 99.74 | 99.74 | 98.21 | 98.21 | 100 |  |  |  |  |  |  |  |  |  |  |  |  |  |
| N1 | 98.68 | 98.21 | 98.21 | 98.19 | 99.74 | 99.74 | 98.17 | 100 |  |  |  |  |  |  |  |  |  |  |  |  |
| N2 | 98.68 | 98.21 | 98.21 | 98.19 | 99.74 | 99.74 | 98.17 | 99.98 | 100 |  |  |  |  |  |  |  |  |  |  |  |
| E1 | 98.83 | 98.11 | 98.11 | 98.09 | 99.10 | 99.10 | 98.05 | 99.06 | 99.06 | 100 |  |  |  |  |  |  |  |  |  |  |
| A1 | 99.10 | 98.22 | 98.22 | 98.20 | 99.22 | 99.22 | 98.16 | 99.16 | 99.16 | 99.52 | 100 |  |  |  |  |  |  |  |  |  |
| A2 | 99.03 | 98.22 | 98.22 | 98.20 | 99.23 | 99.23 | 98.16 | 99.18 | 99.18 | 99.53 | 99.81 | 100 |  |  |  |  |  |  |  |  |
| A3 | 99.02 | 98.21 | 98.21 | 98.19 | 99.22 | 99.22 | 98.15 | 99.17 | 99.17 | 99.53 | 99.80 | 99.99 | 100 |  |  |  |  |  |  |  |
| A4 | 99.08 | 98.20 | 98.20 | 98.18 | 99.20 | 99.20 | 98.14 | 99.14 | 99.14 | 99.50 | 99.98 | 99.79 | 99.79 | 100 |  |  |  |  |  |  |
| A5 | 99.10 | 98.22 | 98.22 | 98.20 | 99.21 | 99.21 | 98.16 | 99.15 | 99.15 | 99.51 | 99.99 | 99.80 | 99.79 | 99.97 | 100 |  |  |  |  |  |
| A6 | 99.09 | 98.21 | 98.21 | 98.19 | 99.21 | 99.21 | 98.15 | 99.15 | 99.15 | 99.51 | 99.99 | 99.80 | 99.79 | 99.97 | 99.98 | 100 |  |  |  |  |
| A7 | 99.09 | 98.21 | 98.21 | 98.19 | 99.21 | 99.21 | 98.15 | 99.15 | 99.15 | 99.51 | 99.99 | 99.80 | 99.79 | 99.97 | 99.98 | 99.98 | 100 |  |  |  |
| A8 | 99.09 | 98.21 | 98.21 | 98.19 | 99.21 | 99.21 | 98.15 | 99.15 | 99.15 | 99.51 | 99.99 | 99.80 | 99.79 | 99.97 | 99.98 | 99.98 | 99.98 | 100 |  |  |
| A9 | 99.09 | 98.21 | 98.21 | 98.19 | 99.21 | 99.21 | 98.15 | 99.15 | 99.15 | 99.53 | 99.99 | 99.80 | 99.79 | 99.97 | 99.98 | 99.98 | 99.98 | 99.98 | 100 |  |
| A10 | 99.09 | 98.21 | 98.21 | 98.19 | 99.21 | 99.21 | 98.15 | 99.15 | 99.15 | 99.51 | 99.99 | 99.80 | 99.79 | 99.97 | 99.98 | 99.98 | 99.98 | 99.98 | 99.98 | 100 |
